# Alterations in transcript abundance of bovine oocytes recovered at growth and dominance phases of the first follicular wave

**DOI:** 10.1186/1471-213X-7-90

**Published:** 2007-07-27

**Authors:** Nasser Ghanem, Michael Hölker, Franca Rings, Danyel Jennen, Ernst Tholen, Marc-André Sirard, Helmut Torner, Wilhelm Kanitz, Karl Schellander, Dawit Tesfaye

**Affiliations:** 1 Institute of Animal Science, Animal Breeding and Husbandry Group, University of Bonn, 53115 Bonn, Germany; 2 Centre de Recherche en Biologie de la Reproduction, Université Laval, Département des Sciences Animales, Pav. Comtois, Laval, Sainte-Foy, Québec, G1K 7P4, Canada; 3 Research Institute for Biology of Farm Animals, Wilhelm-Stahl-Allee 2, 18196 Dummerstorf, Germany

## Abstract

**Background:**

Oocyte developmental competence is highly affected by the phase of ovarian follicular wave. Previous studies have shown that oocytes from subordinate follicles recovered at growth phase (day 3 after estrus) are developmentally more competent than those recovered at dominance phase (day 7 after estrus). However, the molecular mechanisms associated with these differences are not well elucidated. Therefore, the objective of this study was to investigate transcript abundance of bovine oocytes retrieved from small follicles at growth and dominance phases of the first follicular wave and to identify candidate genes related to oocyte developmental competence using cDNA microarray.

**Results:**

Comparative gene expression analysis of oocytes from growth and dominance phases and subsequent data analysis using Significant Analysis of Microarray (SAM) revealed a total of 51 differentially regulated genes, including 36 with known function, 6 with unknown function and 9 novel transcripts. Real-time PCR has validated 10 transcripts revealed by microarray analysis and quantified 5 genes in cumulus cells derived from oocytes of both phases. The expression profile of 8 (80%) transcripts (ANAXA2, FL396, S100A10, RPL24, PP, PTTG1, MSX1 and BMP15) was in agreement with microarray data. Transcript abundance of five candidate genes in relation to oocyte developmental competence was validated using Brilliant Cresyl Blue (BCB) staining as an independent model. Furthermore, localization of mRNA and protein product of the candidate gene MSX1 in sections of ovarian follicles at days 0, 1, 3 and 7 of estrous cycle showed a clear fluorescent signal in both oocytes and cumulus cells with higher intensity in the former. Moreover, the protein product was detected in bovine oocytes and early cleavage embryos after fertilization with higher intensity around the nucleus.

**Conclusion:**

This study has identified distinct sets of differentially regulated transcripts between bovine oocytes recovered from small follicles at growth and dominance phases of the first follicular wave. The validation with independent model supports our notion that many of the transcripts identified here may represent candidate genes associated with oocyte developmental competence. Further specific functional analysis will provide insights into the exact role of these transcripts in oocyte competence and early embryonic development.

## Background

In vitro embryo production depends on the collection of immature oocytes from heterogeneous follicular population of slaughterhouse ovaries. Despite the desire to expand the field application of this technology in cattle, the blastocyst development is limited to 30–40% with only half of these being able to initiate a successful pregnancy following transfer [1,2]. One of the main factors affecting the embryo yield is the intrinsic quality of the oocyte, while the embryo culture condition plays a crucial role in determining embryo quality [3].

There is a general agreement that oocyte developmental competence is related to follicule size [4], estrous cycle stage [5] and the level of atresia influenced by other follicles, mainly the dominant follicle [6] and morphological features of the oocyte [7]. Follicle size and oocyte diameter are closely related, and as both increase the developmental potential of the oocyte also increases [8-10]. However, various studies revealed that the developmental competence of an oocyte is related to the status of the follicle from which it is obtained regardless of follicle size [11-13].

As soon as the primordial follicle store is established, follicle recruitment begins and continues in a wave-like pattern during estrous cycles. Bovine estrous cycle is characterized by a series of two or three follicular waves [14-16]. Within several days of initiation of a wave, one follicle is selected as the dominant follicle, which continues to grow and differentiate, whereas its sister subordinate follicles plateau in growth and then regress. The interactions between the follicles throughout each follicular wave affect oocyte quality. It was reported [6,17-21] that development of early embryos to the blastocyst stage was greater when oocytes are obtained during follicular growth/stagnation phase (G/S) than in the dominance/regression phase (D/R). The dominant follicle exerts a direct inhibitory effect on the development of subordinate follicles [22], causing them to undergo atresia [23], which lead to lower in vitro developmental competence compared to their counterparts at growth phase [6]. In addition, blastocysts derived from oocytes collected from both medium and small follicles at G/S stage or D/R stage were reported to be different in relative abundance of transcripts related to embryonic development [24].

To our knowledge, the mRNA transcript abundance of oocytes recovered at different stages of ovarian follicular turnover in bovine has not yet been analyzed. Therefore, in the present study we aimed to identify the differences in the transcript abundance of bovine oocytes retrieved from small follicles at growth and dominance phases which could be associated with oocyte developmental potential. With this approach, we have identified differentially expressed genes in oocytes derived from these two stages. Differences in transcript abundance of some transcripts of interest between the two oocyte groups and cumulus cells were examined using quantitative real-time PCR. An independent model has been used to validate the expression profile of some selected transcripts in oocytes screened for differences in their developmental competence.

In addition, MSX1 gene was selected for further analysis due to the following reasons: 1) it has also been identified as differentially regulated transcript between biopsies derived from blastocysts resulted in pregnancy and no pregnancy in our previous study [25] 2) results from previous studies [26,27] suggest a physiologically optimal level of MSX1 expression is vital for normal cellular function. However, so far this transcript has not been characterized in early embryonic development and follicular turnover. The distribution of protein products and mRNA for MSX1 during ovarian follicular turnover and early embryonic development has been evidenced in this study.

## Results

### Follicle distribution and oocyte recovery

As shown in Table 1, ultrasound guided ovum pick up (OPU) of sixty cyclic heifers showed that ovaries of both growth and dominance phases had greater number of small follicles (3–5 mm) than medium and large follicles. Moreover, a higher number of small and medium follicles (n = 333) has been found at growth phase compared to dominance phase (n = 262). Consequently, the average number of oocytes collected per cow was greater during growth phase (7.7 ± 3.5) than in the dominance phase (4.9 ± 2.1).

**Table 1:** Performance of the OPU procedure at growth and dominance phases of follicular development

Phase of follicular development	Follicle size	Number of follicles aspirated	Number of oocytes retrieved	Average number of oocytes per animal	Recovery rate
Growth (n = 30)	Large	-	-		
	Medium	148	88	7.7 ± 3.5	69.4%
	Small	185	143		
Dominance (n = 30)	Large	30	7		
	Medium	39	20	4.9 ± 2.1	50.7%
	Small	223	121		

### Comparative transcript abundance of oocytes recovered at growth and dominance phases

**Figure 1:**
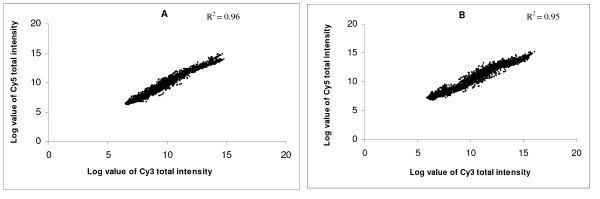
Scatter plot illustrating log value of Cy3, Cy5 total intensities for the biological and dye swap (technical) replicates. Each gene is represented by a point of Cy3 and Cy5 log values, A: represents target hybridizations B: represents dye swap hybridizations.

In the present study, a series of six hybridizations (three biological replicates with dye swaps) were conducted to minimize the false positive expression changes and to identify genes truly differentially expressed between oocytes recovered from growth and dominance phases. The generated data are available on our website of Bonn University [28], under the research section for OPU-IVF. After LOWESS normalization of the data, log value of Cy5 total intensity was compared with the log value of Cy3 total intensity for both the target (Fig. 1A) and the respective dye swap hybridizations (Fig. 1B). The coefficient of determination of the target (R^2 ^= 0.96) and dye swap (R^2 ^= 0.95) hybridizations were found to be relatively the same which confirms that there was low false positive expression changes between hybridizations of differentially labeled (Cy5 and Cy3) cDNA from the same phase (growth and dominance).

**Figure 2:**
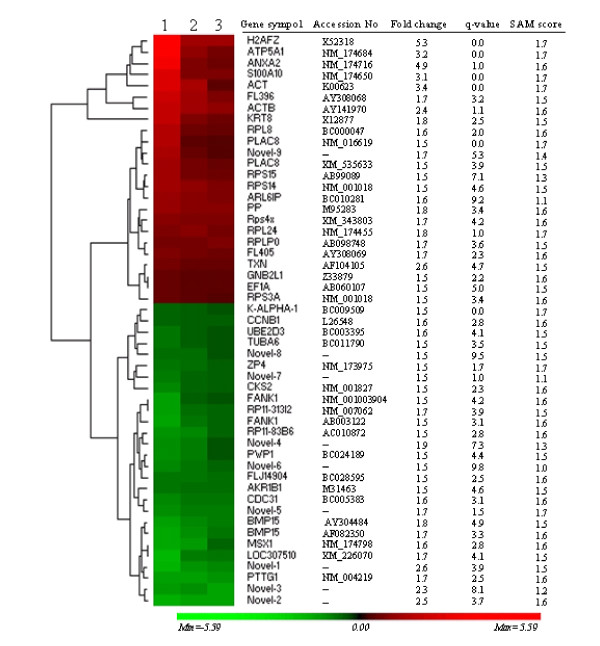
Hierarchical clustering and heatmap of 51 differentially expressed genes. The red blocks represent up-regulated genes while the green blocks represent down-regulated genes in oocytes recovered at growth phase.

To obtain a highly confident set of differentially expressed genes, we used a rigorous combination of P-value (*P *< 0.05) and fold discovery rate (FDR) of 10%. Accordingly, the data analysis performed by SAM showed that 51 transcripts were differentially regulated between the two oocyte populations. The threshold for significant variation was set at 1.5 fold change. Using this criterion, genes were sorted into two categories based on their variation factor: those for which the relative transcript abundance increased (up-regulated) or decreased (down-regulated) in growth phase versus dominance phase oocytes. The fold change was in a range of 1.5 to 5.3 for up-regulated and 1.5 to 2.6 for down-regulated genes in growth phase compared to dominance phase oocytes (Fig. 2, Tab. 2 and 3).

**Table 2:** Transcripts up-regulated in oocytes recovered at growth phase compared to dominance phase

Gene name	Accession No in NBCI GenBank	Fold change	Gene function
Bos taurus beta-actin mRNA complete cds (ACTB)	AY141970	2.4	Structural constituent of cytoskeleton
Bovine actin mRNA 3' end (ACT)	K00623	3.4	Structural constituent of cytoskeleton
Bovine mRNA for histone (H2AFZ)	X52318	5.3	Chromosome organization
Bovine mRNA fragment for cytokeratin A (no. 8) (KRT8)	X12877	1.8	Cytoskeleton organization and biogenesis
Bos taurus S100 calcium-binding protein A10 mRNA (S100A10)	NM_174650	3.1	Calcium ion binding
Bos taurus annexin A2 mRNA (ANXA2)	NM_174716	4.9	Calcium ion binding
Bos taurus ATP synthase, H+ transporting, mitochondrial F1 complex (ATP5A1)	NM_174684	3.2	ATP binding
Bos taurus isolate FL396 mitochondrion partial genome	AY308068	1.7	NADH dehydrogenase (ubiquinone) activity NADH dehydrogenase (ubiquinone) activity
Bos taurus isolate FL405 mitochondrion, partial genome	AY308069	1.7	Structural constituent of ribosome
Bos taurus ribosomal protein L24 mRNA (RPL24)	NM_174455	1.8	Structural constituent of ribosome
Rattus norvegicus ribosomal protein S4, X-linked mRNA (Rps4x)	XM_343803	1.7	Structural constituent of ribosome
Homo sapiens ADP-ribosylation factor-like 6 interacting protein (ARL6IP)	BC010281	1.6	Structural constituent of ribosome
Bos taurus mRNA for similar to acidic ribosomal phosphoprotein PO (RPLP0)	AB098748	1.7	Structural constituent of ribosome
Homo sapiens ribosomal protein L8, mRNA (cDNA clone IMAGE:3504599) (RPL8)	BC000047	1.6	Structural constituent of ribosome
Bos taurus mRNA for similar to ribosomal protein S3a, partial cds (RPS3A)	AB099017	1.5	Structural constituent of ribosome
Bos taurus mRNA for similar to ribosomal protein S14, partial cds (RPS14)	AB099089	1.5	Structural constituent of ribosome
Homo sapiens ribosomal protein S15 mRNA (RPS15)	NM_001018	1.5	Translation elongation factor activity
Bos taurus mRNA for elongation factor 1 alpha, complete cds (EF1A)	AB060107	1.5	Inorganic diphosphatase, Phosphatase
Bovine inorganic pyrophosphatase mRNA sequence (PP)	M95283	1.8	Thiol-disulfide exchange intermediate activity
Bos taurus thioredoxin mRNA complete cds (TXN)	AF104105	2.6	Signal transduction
S.scrofa mRNA encoding G-beta like protein (GNB2L1)	Z33879	1.5	Unknown
Homo sapiens placenta-specific 8 mRNA (PLAC8)	NM_016619	1.5	Unknown
PREDICTED: Canis familiaris similar to Placenta-specific gene 8 ((PLAC8)	XM_535633	1.5	

Differentially expressed genes were identified by SAM at a false discovery rate (FDR) of ≤ 10% and *P *< 0.05.

**Table 3:** Transcripts down-regulated in oocytes recovered at growth phase compared to dominance phase

Gene name	Accession No in NBCI GenBank	Fold Change	Gene function
Bos taurus bone Morphogenetic protein 15 mRNA partial cds (BMP15)	AY304484	1.8	Growth factor activity
Homo sapiens bone morphogenetic protein 15 precursor gene (BMP15)	AF082350	1.7	Growth factor activity
Bos taurus msh homeo box homolog 1 (Drosophila) mRNA (MSX1)	NM_174798	1.6	Transcription factor activity
Homo sapiens pituitary tumor-transforming gene 1 mRNA (PTTG1)	NM_004219	1.7	Transcription factor activity
Homo sapiens fibronectin type 3 and ankyrin repeat domains 1 mRNA (FANK1)	BC024189	1.5	Transcription factor activity
Bos taurus fibronectin type 3 and ankyrin repeat domains 1 (FANK1)	NM_001003904	1.5	Transcription factor activity
Homo sapiens nuclear phosphoprotein similar to S. cerevisiae (PWP1)	NM_007062	1.5	Cell growth and/or transcription
Homo sapiens CDC28 protein kinase regulatory subunit 2 mRNA (CKS2)	NM_001827	1.5	Cell cycle
Bos taurus mRNA sequence (CCNB1)	L26548	1.6	Regulation of cell cycle progression
Homo sapiens centrin, EF-hand protein, 3 (CDC31 homolog, yeast)	BC005383	1.6	Mitotic centrosome separation
Homo sapiens ubiquitin-conjugating enzyme E2D 3 (UBC4/5 homolog) (UBE2D3)	BC003395	1.6	Ubiquitin conjugating enzyme activity
Homo sapiens tubulin, alpha, ubiquitous, mRNA (cDNA clone MGC:4689) (K-ALPHA-1)	BC009509	1.5	Nucleotide binding
Homo sapiens tubulin alpha 6, mRNA (cDNA clone MGC:19827) (TUBA6)	BC011790	1.5	Nucleotide binding
Bovine aldose reductase mRNA 3' end (AKR1B1)	M31463	1.5	Aldehyde reductase activity
Bos taurus zona pellucida glycoprotein 4 mRNA (ZP4)	NM_173975	1.5	Binding of sperm to zona pellucida
Rattus norvegicus similar to putative nuclear protein (LOC307510)	XM_226070	1.7	Unknown
Homo sapiens genomic DNA, chromosome 11q, (clone:RP11-313I2)	AP003122	1.7	Unknown
Homo sapiens BAC clone RP11-83B6 from 2, complete sequence (RP11-83B6)	AC010872	1.5	Unknown
Homo sapiens hypothetical protein FLJ14904, mRNA (cDNA clone) (FLJ14904)	BC028595	1.5	Unknown

Differentially expressed genes were identified by SAM at a false discovery rate (FDR) of ≤ 10% and *P *< 0.05.

Hierarchical clustering and heatmap of differentially regulated genes showed the overall expression pattern between the two oocyte groups. The average linkage clustering analysis revealed the presence of many subgroups (or clusters) within the up- and down-regulated genes sharing similar expression pattern (Fig. 2).

### Functional classification of differentially regulated genes

**Figure 3:**
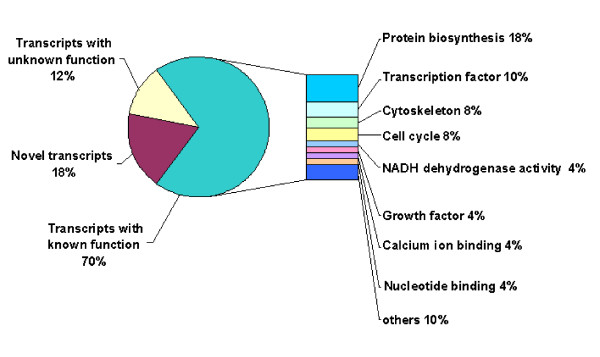
Differentially expressed genes as classified based on the Gene Ontology Consortium classifications [29].

All differentially regulated transcripts were functionally classified based on the criteria of Gene Ontology Consortium classifications [29]. The resulting data were supplemented with additional information from various tools available in National Center for Biotechnology Information [30]. Accordingly, the differentially regulated transcripts represent genes with known function (36/51), genes with unknown function (6/51) and novel transcripts (9/51) (Fig. 3). Regarding the molecular function, transcripts with known function showed to be involved in protein biosynthesis (18%), transcription (10%), cytoskeleton (8%), cell cycle (8%), NADH dehydrogenase activity (4%), calcium ion binding (4%), nucleotide binding (4%) and other functions (10%). Oocytes recovered at growth phase (Tab. 2) were enriched with genes regulating protein biosynthesis (RPLP0, RPL8, RPL24, ARL6IP, RpS14, RpS15, RpS4x and RPS3A), translation elongation (EF1A), ATP binding (ATP5A1), NADH dehydrogenase activity (FL396 and FL405), cytoskeleton (Actin, beta-Actin, H2AZ and KRT8), calcium ion binding (S100A10 and ANXA2), signal transduction (G-beta like protein), (.)inorganic diphosphatase (PP) and thiol-disulfide exchange intermediate (TXN). On the other hand, oocytes recovered at dominance phase (Tab. 3) were encoded transcripts controlling transcription (MSX1, PTTG1, FANK1 and PWP1), cell cycle (CCNB1, CKS2, UBE2D3 and CDC31), aldehyde reductase activity (AKR1B1), nucleotide binding (TUBA6 and K-ALPHA-1), growth factor (BMP15) and fertilization (ZP4).

### Real-time PCR Validation

**Figure 4:**
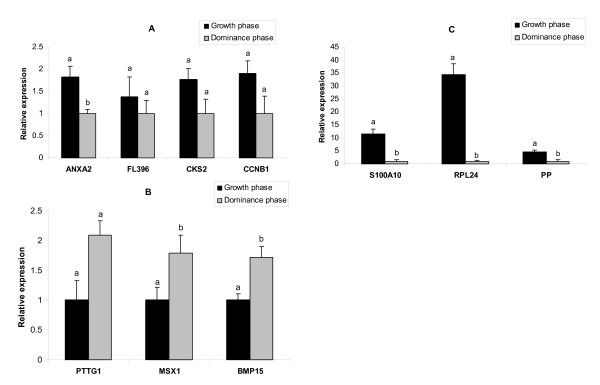
Quantitative real-time PCR validation of 10 differentially expressed genes in bovine oocytes recovered at growth phase (day 3) vs. dominance phase (day 7) as identified by microarray analysis (A, B, C). The relative abundance of mRNA levels represents the amount of mRNA compared to the calibrator (with the lowest normalized value). Bars with different superscripts (a, b) are significantly different at *P *< 0.05.

Real-time PCR analysis using independent oocyte samples was conducted to validate the array results of 10 differentially regulated transcripts. No differences in relative abundance of the internal control gene (GAPDH) were observed in all samples. Based on this, the quantitative real-time PCR has confirmed the relative abundance of 8 transcripts (80%) to be in agreement with microarray results (Fig. 4A, B, C). The mRNA relative abundance of mRNAs for ANAXA2, S100A10, RPL24, and PP was higher (*P *< 0.05) in growth phase compared to dominance phase oocytes. Greater transcript abundance for MSX1 and BMB15 was observed in dominance phase versus growth phase oocytes. However, differences in transcript abundance of FL396 and PTTG1 between the two oocyte groups were not statistically significant. The array results of CKS2 and CCNB1 could not be confirmed by real-time quantitative PCR (Fig. 4A).

### Quantitative analysis of selected transcripts in cumulus cells

**Figure 5:**
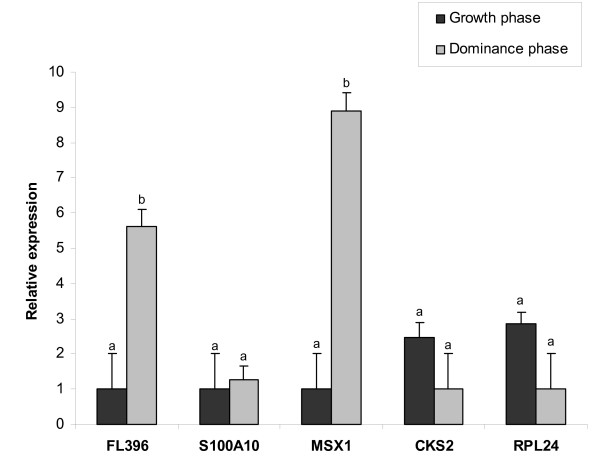
Quantitative real-time PCR of 5 genes in bovine cumulus cells denuded from oocytes recovered at growth phase (day 3) vs. dominance phase (day 7). The relative abundance of mRNA levels represents the amount of mRNA compared to the calibrator (with the lowest normalized value). Bars with different superscripts (a, b) are significantly different at *P *< 0.05.

To avoid any biasness in the number of cumulus cells used as an input for mRNA isolation and subsequent real-time PCR, cumulus cells were derived from equal number of cumulus oocyte complexes (COCs) with similar morphological appearance. After mRNA isolation and subsequent cDNA synthesis, the relative abundance of GAPDH gene was tested and showed no variation between these samples. Two transcripts (MSX1 and FL396) were highly abundant (*P *< 0.05) in cumulus cells of dominance phase than that of growth phase (Fig. 5). On the other hand, RPL24 and CKS2 transcripts were found to be more abundant in cumulus cells of growth phase compared to that of dominance phase. The relative abundance of mRNA for S100A10 was nearly the same in cumulus cells of both phases.

### Validation of relationship between transcript abundance and oocyte competence using BCB staining

**Figure 6:**
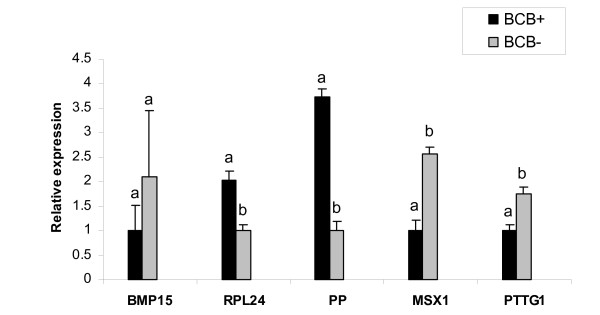
Quantitative real-time PCR of 5 genes in bovine oocytes treated with brilliant cresyl blue stain [BCB- (colourless cytoplasm, increased G6PDH activity) and BCB+ (coloured cytoplasm, low G6PDH activity)]. The relative abundance of mRNA levels represents the amount of mRNA compared to the calibrator (with the lowest normalized value). Means without common superscripts (a, b) were considered to be significantly different at *P *< 0.05.

Immature oocytes screened for developmental competence based on BCB staining were used for validation of the expression profile of five transcripts (BMP15, RPL24, PP, MSX1 and PTTG1). The cDNA synthesized from BCB^+ ^(stained blue) and BCB^- ^(stained colourless) oocyte samples were quantified using real-time PCR. After confirming that there were no significant differences in the relative abundance of GAPDH between the samples, all transcripts were quantified using independent real-time PCR runs. The relative abundance for MSX1 and PTTG1 were higher (*P *< 0.05) in BCB^- ^oocytes than BCB^+ ^ones (Fig. 6). The BMP15 was also more abundant in BCB^- ^than BCB^+ ^oocytes but differences were not significant. On the other hand, greater mRNA abundance (*P *< 0.05) for RPL24 and PP has been observed in BCB^+ ^than BCB^- ^oocytes (Fig. 6).

### Localization of bovine MSX1 mRNA and protein

**Figure 7:**
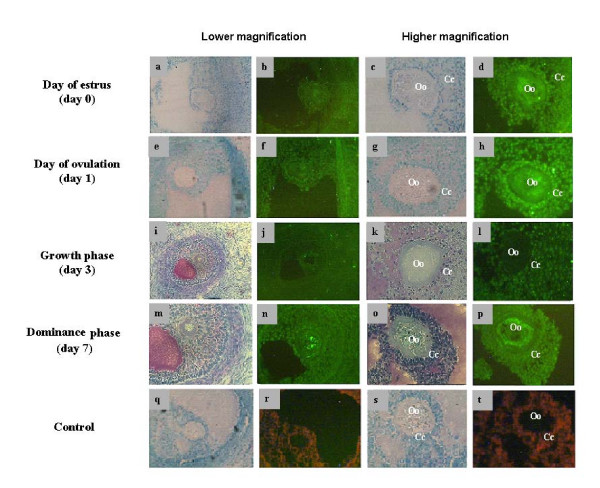
Immunohistochemical localisation of MSX1 protein in bovine ovarian sections at day of estrus (b, d), day of ovulation (f, h), growth phase (j, l), dominance phase (m, q). Cumulus cells are marked with Cc and oocytes are marked with Oo. Negative controls were processed without addition of primary anti-MSX1 antibody (r, t). Sections were counterstained with toluidine blue (a, c, e, g, i, k, m, o, q and s). Images from the same ovarian sections were captured with lower and higher magnification.

**Figure 8:**
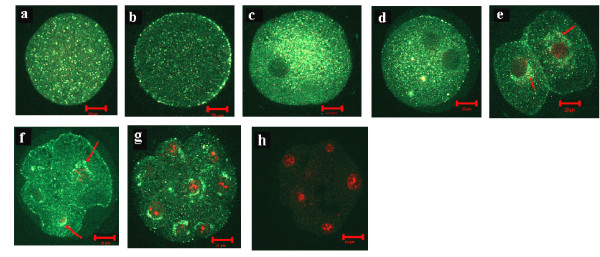
Subcellular localization of bovine MSX1 protein in bovine oocytes and early cleavage embryonic stages [immature oocyte (a), matured oocyte (b), zygote (c), advanced zygote (d), 2-cell (e), 4-cell (f) and 8-cell (g)]. Negative control (h) was processed without addition of primary anti-MSX1 antibody. Nuclei are stained with propidium iodide (red). Scale bars represent 20 μm.

**Figure 9:**
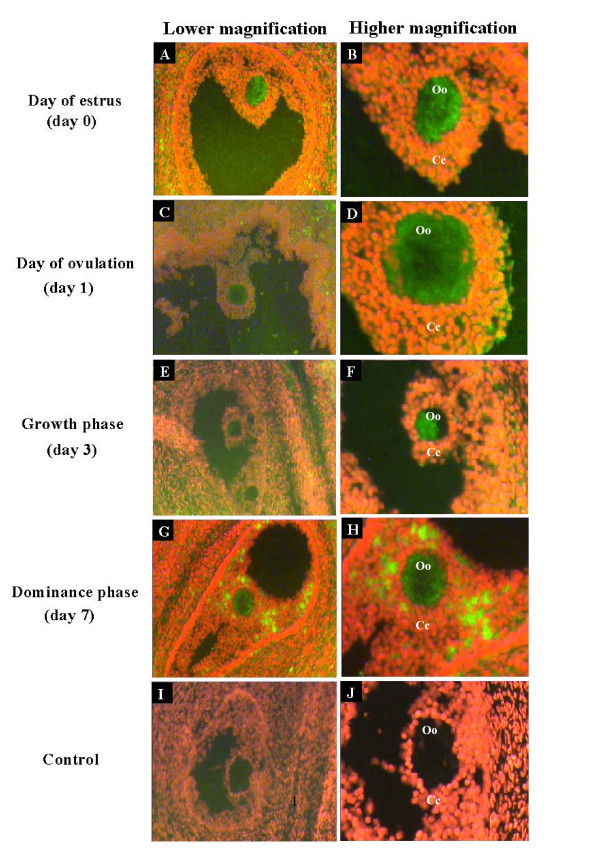
Fluorescent in situ hybridization of MSX1 mRNA conducted with DIG labelled RNA antisense probe in bovine ovarian sections at day of estrus (A, B), day of ovulation (B, C), growth phase (D, E), dominance phase (F, G). Cytoplasms of the oocytes (Oo) are darkly stained with green fluorescent compared to cumulus cells (Cc). Negative controls were hybridized with DIG labelled RNA sense probe (H, I). Images from the same ovarian sections were captured with lower and higher magnification.

Immunofluorescent labelling of MSX1 gene in ovarian samples collected at the time of estrus (day 0), ovulation (day 1), growth phase (day 3) and dominance phase (day 7) showed the presence of this protein in all stages of follicular development under investigation. More specifically, this protein was found to be more localized in the oocyte cytoplasm than the enclosed cumulus cells (Fig. 7d, h, p) or other cellular layers of the growing follicle (Fig. 7b, f, n) at all stages of follicular development except at growth phase (Fig. 7j, l). Furthermore, MSX1 protein was found to be dispersed in the cytoplasm of immature and matured oocytes and early zygote (Fig. 8a, b, c) but tends to be localized around the nucleus at advanced zygote, 2-cell, 4-cell and 8-cell embryos (Fig. 8d, e, f, g). Comparative analysis of protein signals between oocytes showed that green fluorescence signals were reduced after maturation (Fig. 8b). Moreover, in situ hybridization experiment showed that MSX1 mRNA was localized in the oocytes, cumulus cells and follicular wall throughout all stages of follicular turnover (Fig. 9A, B, C, D, E, F, G, H).

## Discussion

Investigation on the molecular characteristics of oocytes of varying developmental competence is critical for the development of future classification criteria for the selection of oocytes with superior developmental capacity [31]. It has been shown that the molecular causes for poor developmental capacity of bovine oocytes may be highly complex and may be reliant on many small changes in the RNA levels of many genes [32]. However; our understanding of composition of the oocyte transcriptome and the identity of key oocyte-expressed genes with important regulatory roles in folliculogenesis and early embryonic development is far from complete [33]. Thus, our study mainly focuses on identifications of differences in mRNA transcript abundance which may be associated with developmental competence of bovine oocytes using cDNA microarray as a functional genomics approach.

Growing oocytes have higher rate of transcription and translation resulting in the formation of RNAs and proteins both for oocyte growth and storage [34]. The previous molecules could be redirected to fulfil new tasks as resumption of meiosis [35] and regulating maternal to zygotic transition [36]. Developmental competence of mammalian oocytes depends on high rates of RNA and protein synthesis, imprinting processes and biogenesis of organelles such as mitochondria [37]. Similarly, in the present study oocytes from the growth phase were found to be enriched with transcripts related to protein biosynthesis as compared to their dominance phase counterparts. An adequate pool of ribosomes, which were synthesized by transcription of the ribosomal RNA (rRNA) genes, is required for protein synthesis during oocyte growth and embryonic development. Failure to detect one or all of ribosomal RNA (rRNA) genes during oocyte growth or later during interphase of the first cell cycle is an indicator of developmental incompetence [38]. This is in agreement with our results that showed the down-regulation of RPL24 transcript in oocytes from dominance phase with low developmental potential and could be validated using BCB screened oocytes. This may suggest that there is an optimal threshold of mRNA transcript abundance for ribosomal RNA genes below which the developmental competence of the oocytes is compromised.

Various transcription factors are known to be present in the maternal mRNA store until their recruitment for translation at the time of maternal-embryonic transition [39]. In the present study, different transcription factors (MSX1, PTTG1, FANK1 and PWP1) were found to be up-regulated in dominance phase compared to growth phase oocytes. MSX gene families encode homeodomain transcription factors [40] and their protein products control key developmental processes such as differentiation and patterning during early development [41]. Overexpression of the MSX1 suppresses cell growth and cell cycle progression in human ovarian cancer cell line by regulating the expression of key cell cycle regulators [42]. In addition, MSX1 has been reported to regulate the p53 tumor suppressor protein in human tumours and thereby enhances apoptosis mediated suppression of p53 [43]. Moreover, mice MSX1 homozygous mutants die at birth [44]. In a recent study, MSX1 was more abundant in oocytes with reduced development competence [31].

Pituitary tumor transforming gene (PTTG1) was isolated from a pituitary tumor cell line and its over-expression results in cellular transformation in vitro and tumor formation in nude mice [45]. Moreover, this gene stimulates expression of the Bax gene, which induces apoptosis in human embryonic kidney cell line [46]. Consistent with the previous facts, the enrichment of dominance phase oocytes with apoptotic related genes (MSX1 and PTTG1) may be related to the level of atresia in subordinate follicles as influenced by the dominant follicle which may lead to reduced developmental capacity of enclosed oocytes. In a recent study conducted in our laboratory, MSX1 and PTTG1 were found to be up-regulated in embryo biopsies derived from blastocysts which resulted in no pregnancy after transfer to recipients [25].

Optimal expression of MSX1 as transcription factor seems to be important for cellular physiology [26,27]. In the present study, the MSX1 protein was detected in all follicular compartments of the growing follicle at different stages of follicular turnover and early preimplantation stages, with a slight decrease in fluorescent intensity in ovaries at growth phase. As embryonic development proceeds there is accumulation of the MSX1 protein around the nucleus of the blastomeres which is consistent with the localization of homeobox gene family namely cdx2 in mouse embryos [47]. Furthermore, mRNA for MSX1 was also distributed in all follicular layers with intensive signal in the oocytes. As a member of transcription factor gene, the possible role of MSX1 in regulating follicular turnover and early embryonic development needs further investigations.

Oocyte growth during folliculogenesis is regulated by granulosa cell derived proteins [48], which are in turn regulated by oocyte-derived factors [49]. In addition, cumulus cells provide nutrients for the oocyte and influence oocyte development in a paracrine fashion [50], and these paracrine factors contribute to induce meiotic resumption of oocytes [51]. Characterization of the signalling pathways driving changes in transcript abundance for co-regulated and differentially regulated genes in oocytes versus associated cumulus cells may lead to a better understanding of interdependent gene regulation between germ and somatic cells [52]. In this study, compared to other candidate transcripts the bovine MSX1 was found to be higher in both oocytes and the corresponding cumulus cells at dominance phase than those from growth phase. Therefore, this transcript can be considered as one of the factors activated in oocyte and cumulus cells from the subordinate follicles due to the presence of the dominant follicle. Similar studies [53,54] have described the expression profile of some transcripts (cyclooxygenase-2, hyaluronic acid synthase-2, gremlin, and pentraxin-3) in the cumulus cells as potential markers of the quality of the enclosed oocyte.

The BCB staining was used effectively to select bovine oocytes for further developmental competence after in vitro fertilization [55] or somatic cell nuclear transfer [56]. Therefore, in the present study, BCB staining was used as an independent model to screen oocytes for their developmental competence in order to validate the differential expression of candidate genes identified in array analysis. Higher abundance of MSX1 and PTTG1 in dominance phase oocytes in array experiment was consistent with their higher abundant in BCB^- ^oocytes than in BCB^+ ^ones. Similarly, the relatively higher abundance of RPL24 and PP transcripts in oocytes from growth phase was consistent with their higher abundance in BCB^+ ^oocytes. Thus, there is a clear association of mRNA abundance for genes detected in microarray experiment and developmental competence of oocyte tested with BCB staining. The bovine RPL24 and MSX1 as members of ribosomal proteins and transcription factor gene family, respectively can be considered as good markers of oocyte developmental competence, as they showed consistent results in both models.

## Conclusion

The reported differences in developmental competence of bovine oocytes derived from small follicles at growth and dominance phases of follicular development are also accompanied by differences in the relative abundance of transcripts related to the various molecular events and processes regulating oocyte competence and follicular development. Our validation with an independent model for the relative abundance of some selected transcripts, supports the notion that many of the transcripts identified as differentially regulated and described here may represent marker candidate genes for oocyte developmental competence. However, the exact role of these transcripts in controlling oocyte developmental potential needs further investigation.

## Methods

### Experimental animals, estrus synchronization and detection

The experimental protocol was carried out according to the rules and regulations of the German law of animal protection.

Sixty Simmental cyclic heifers 24 to 30 months old, were selected based on general clinical examination and normal ovarian cyclicity as determined by ultrasound scanning. The animals were housed as one group and were fed grass silage ad libitum. Estrus and subsequent ovulation were synchronized by two administrations of prostaglandin PGF2α (Estrumate, 2 ml i.m, Fa. Essex, Germany) at 11 days interval followed by GnRH injection (Receptal, 2500 IU i.m, Intervet, Unterschleissheim, Germany) on the day of estrus onset. Estrus was detected after two days of the last prostaglandin PGF2α treatment. Common signs of estrus were monitored by visual observation followed by careful palpation of ovaries for confirmation.

### Ovum pick up (OPU)

OPU was performed at two different phases of follicular development during the first follicular wave using a total of 60 heifers at growth phase (day 3 after estrus, n = 30) and dominance phase (day 7 after estrus, n = 30) for two sessions of OPU in each phase. The heifers were restrained in a chute, and given 5 ml of epidural anesthesia (procaine hydrochloride 2%, Selectavet, Munich, Germany). Using transvaginal ultrasound scanner (Pie Medical 400 Vet, Maastricht, Netherlands) with a 7.5 MHz sector probe the follicles were visualized on the monitor and counted. The follicles were classified according to their size into three categories: small (3–5 mm), medium (6–8 mm) and large follicles (≥ 9 mm). A 50-cm-long 18-gauge needle was passed through a needle guide along the polyethylene housing of the transducer, and carried into the fornix vagina. After fixing the ovary against the transducer, the needle was advanced to puncture the vaginal wall to enter the ovarian follicle. The needle was attached via Teflon tubing to a 50-ml Falcon tube and vacuum pressure provided with a regulated vacuum pump (K-MAR-5000B; William Cook Europe) and adjusted to create a flow rate of 16–20 ml/min. The follicular content of each heifer was aspirated individually into modified Parker maturation medium (MPMM) supplemented with 15% oestrus cow serum (OCS), 0.5 mM L-glutamine, 0.2 mM pyruvate, 50 μg/ml gentamycin sulphate, 10 μl/ml FSH (Folltropin, Vetrepharm, Canada) and kept at 39°C in thermos. The follicular fluid contents were poured into a square grid dish to facilitate finding of oocytes under a stereomicroscope. The collected oocytes were kept in the same maturation medium used for collection before being separated from the surrounding cumulus cells. Oocytes from each follicular size category and phase were handled and frozen separately.

### Oocytes denudation and storage

In this study, only COCs from the small follicles of the growth and dominance phases were used for the analysis of transcript abundance. Cumulus cells were removed from the oocytes of each phase mechanically in maturation medium supplemented with hyaluronidase 1 mg/ml (Sigma). Separation of cumulus cells was carefully checked under a stereomicroscope. Cumulus free oocytes and the corresponding cumulus cells of each phase were washed two times in PBS (Sigma) and snap frozen separately in cryo-tubes containing 20 μl of lysis buffer [0.8 % IGEPAL (Sigma), 40 U/μl RNasin (Promega Madison WI, USA), 5 mM dithiothreitol (DTT) (Promega Madison WI, USA)]. Finally, samples were stored at -80°C until RNA extraction.

### RNA isolation from oocytes and cumulus cells

Messenger RNA isolation of oocytes and cumulus cells was performed at four different points during the whole experiment. 1) A total of six pools, each containing 20 oocytes from growth and dominance phases of follicular development, were used for array analysis after amplification, 2) A total of six pools, each containing 20 oocytes from growth and dominance phases, were used for real-time validation of array results, 3) The cumulus cells detached from the oocytes at the growth and dominance phases were also used for mRNA isolation and subsequent expression analysis of selected transcripts, 4) A total of 8 pools of oocytes, each containing 50 oocytes from BCB^+ ^and BCB^- ^categories were used to validate the expression profile of selected transcripts in oocytes with different developmental competences. In all cases, mRNA isolation was performed using Dynabead oligo (dT)25 (Dynal Biotech, Oslo, Norway) according to manufacturer's instructions. Briefly, oocytes or cumulus cells in lysis buffer were mixed with 40 μl binding buffer [20 mM Tris HCl with pH 7.5, 1 M LiCl, 2 mM EDTA with pH 8.0] and incubated at 70°C for 5 min to obtain complete lysis of and to release RNA. Ten microlitres of oligo (dT)25 attached magnetic bead suspension were added to the samples, and incubated at room temperature for 30 min. The hybridized mRNA and magnetic beads were washed three times using washing buffer (10 mM Tris HCL with pH 7.5, 0.15 mM LiCl, 1 mM EDTA with pH 8.0). For each sample, cDNA synthesis has been performed using oligo (dT)23 primer and superscript reverse transcriptase II (Invitrogen, Karlsruhe, Germany) except for samples used in array analysis where the reverse transcription was performed using T7 promotor attached oligo d(T)21 primer.

### RNA amplification

First and second-strand cDNA synthesis were carried out as described in our previous study [25]. Ten amplification cycles were used during second strand synthesis as this showed less bias on the representativeness of the original mRNA population after in vitro transcription [57]. The cDNA was purified and used for in vitro transcription using AmpliScribe T7 transcription kit (Epicentre technologies, Oldendorf, Germany) according to manufacturer's instructions. Then the amplified RNA (aRNA) was purified using RNeasy Mini kit (Qiagen, Hilden, Germany) according to the manufacturer's recommendations. Finally, the aRNA was eluted in 30 μl RNase free water from which 8 μl was taken to estimate the yield, purity of aRNA by gel electrophoresis and UV absorbance reading at A260/280 using Ultrospec™ 2100 pro UV/Visible Spectrophotometer (Amersham Bioscience, Freiburg, Germany).

### Aminoallyl indirect labelling and dye coupling

MIAME (Minimum Information About Microarray Experiments) guidelines were adhered to the experimental design. Two independent labelling reactions were carried out per aRNA sample pertinent to each biological replicate for dye-swap hybridizations. Accordingly, 3 μg of aRNA from each oocyte pool representing each follicular phase of development (growth or dominance) was used as template in reverse transcription reactions incorporating amino-modified dUTPs into the cDNA using the CyScribe Post-Labelling Kit (Amersham Biosciences, Freiburg, Germany) as described previously [25,57]. The aminoallyl labelled cDNA samples were purified using CyScribe™ GFX™ Purification kit (Amersham Biosciences) after adding 10 μl of 2 M HEPES. The purified Aminoallyl labelled cDNA was then eluted in 60 μl 0.1 M sodium bicarbonate. The cDNA samples from each phase were differentially labelled indirectly using N-hydroxysuccinate-derived Cy3 and Cy5 dyes and incubated for 1.5 hrs at room temperature in dark. At the end of incubation, non reacting dyes were quenched by adding 15 μl of 4 M hydroxylamine solution (Sigma) and incubated for 15 min at room temperature in dark. To avoid variation due to dye coupling, aRNA samples from the same follicular phase were labelled reversibly either with Cy3 or Cy5 for dye swaps hybridizations. The reaction was then purified with CyScribe™ GFX™ Purification kit (Amersham Biosciences, Freiburg, Germany). Samples were finally eluted in 60 μl elution buffer.

### Probe hybridization

Pre-hybridization of the slides was performed by placing the array slides into a corning GAPS II slide container as described in El-Sayed et al. [25]. Hybridization and post-hybridization washes were carried out as previously described elsewhere [58] with slight modifications. Samples that were going to be hybridized on specific array were mixed and dried in speedvac centrifuge (GMI, Inc. Minnesota, USA) then the pellet was re-suspended in pre-warmed (42°C) formamid based hybridization buffer [15 μl hybridization buffer (Amersham Bioscience, Freiburg, Germany), 30μl 100% Formamide, and 15μl DEPC water]. Yeast tRNA (4 mg/ml) and 2.5 μl of Cot-human DNA (1 mg/ml) (Invitrogen, Karlsruhe, Germany) were added in a volume of 2.5 μl each to avoid non specific hybridisation. The pellet was denatured at 95°C for 5 min, centrifuged briefly and hybridized to the array. The arrays were covered with glass cover slips (ROTH, Karlsruhe, Germany) and fixed in the hybridisation cassettes (TeleChem International, Inc, CA, USA.) before incubation in a hybridisation chamber (GFL, Dülmen, Germany) at 42°C for 16–20 hrs. After hybridization, slides were washed twice with 2× SSC-0.1% SDS buffer for 5 min at 42°C, then once with 1× SSC, 0.2× SSC and 0.1× SSC for 5 min each at room temperature. Finally, the slides were rinsed in RNA free water then in isopropanol for 1 min each and centrifuged at ≥ 2000 rpm for 2 min.

### Custom array characterization

Ready made bovine cDNA array (BlueChip) [59] provided by Centre de Recherche en Biologie de la Reproduction was used in this study. The glass slide contains 4928 spots divided into two sub-arrays. Each sub-array was composed of 2304 ESTs randomly selected clones obtained from four different subtraction suppressive hybridizations (SSH) made with bovine embryos and tissues (First SSH: GV oocytes subtracted from somatic tissues, second SSH: GV oocytes subtracted from day-8 blastocysts, third SSH: day-8 blastocysts subtracted from GV oocytes and fourth SSH: day-8 blastocysts subtracted from somatic tissues). All the clones were spotted in each sub-array for a total of four replicates per slide. Eleven more samples namely vide (32 spots), alien1 (8 spots), alien2 (8 spots), GFP (4 spots), GFP1 (4 spots), GFP1/2 (4 spots), GFP1/4 (4 spots), GFP1/8 (4 spots), GFP 1/16 (4 spots) and H20/DMSO (50 spots) were spotted to be used as negative controls for determination of hybridisation background during the statistical analysis. Housekeeping genes including tubulin (8 spots), ubiquitin (8 spots), β-actin (6 spots) and actin (8 spots) were also added as positive controls.

### Array scanning and data analysis

The slides were scanned using Axon GenePix 4000B scanner (Axon Instruments, Foster City, CA, USA). The GenePix^® ^Pro 4.0 software (Axon Instruments, CA, USA) was used to process the images, to find spots, to integrate robot-spotting files and finally to create reports of spot intensity data. The LOWESS normalization of microarray data was performed using GProcessor 2.0a software [60]. The normalised data were used to calculate intensity ratios of all replicates and to obtain one value per clone. Ratios were finally log_2 _transformed and submitted to SAM analysis. Microarray data analysis was performed using SAM, free software developed at Stanford University [61]. To get truly differentially expressed genes, the FDR was set at 10% and P-value of < 0.05. Hierarchical clustering and heatmap of log_2_-transformed data for up and down regulated genes were generated using PermutMatrix (version 1.8.2) available at [62]. In addition, average linkage clustering algorithm method was employed [63]. Genes expressed equally in both samples were not included in the hierarchical clustering.

### Quantitative real-time PCR analysis

To validate the results obtained from microarray analysis, 10 candidate transcripts were selected for further analysis using real-time quantitative PCR (Tab. 4). Five of these 10 candidates were further quantified in the corresponding cumulus cells from the oocytes of growth and dominance phases of follicular development. Furthermore, five transcripts were subjected to real-time quantitative PCR using cDNA synthesized form BCB^+ ^and BCB^- ^oocytes. In all cases, quantitative analysis of cDNA samples was performed as described previously [25] in comparison with the bovine GAPDH gene (endogenous control), and was run in separate wells using ABI PRISM^® ^7000 sequence detection system (Applied Biosystems, Foster City, CA, USA). Finally quantitative analysis was done using the relative standard curve method and results were reported as the relative expression or fold change as compared to the calibrator after normalization of the transcript level to the endogenous control [64].

**Table 4:** Details of the primers used for real-time quantitative PCR analysis and in situ hybridization

Gene name	Gene bank accession Number	Primer sequences	Annealing temperature (°C)	Product size (bp)
BMP15^a^	AY304484	F: 5'- CTGACGCAAGTGGACACCCTA -3'	60	396
		R: 5'- GACACACGAAGCGGAGTCGTA -3'		
PTTG1^a^	NM_004219	F: 5'- GAAGAGCACCAGATTGCGC -3'	55	204
		R: 5'- GTCACAGCAAACAGGTGGCA -3'		
MSX1^a^	NM_174798	F: 5'- AAGGTATCCACAGTCCCCAGC -3'	55	180
		R: 5'- TCTGCCTCTCCTGCAAAGTTC -3'		
PP^a^	AF170490	F: 5'- GCTGCATCCTACTTGTCGGAA-3'	55	194
		R: 5'- TTCCAAACTACAACCGCCTTG -3'		
S100A10^a^	NM174681	F: 5'- GGATTTCTGAGCATATGGGACC -3'	55	131
		R: 5'- GAGCAAGAGGATGCAAGCAATA -3'		
ANXA2^a^	NM_174716	F: 5'- CGTGCTCCAGCTAACAGTTCT-3'	55	139
		R: 5'- GGAAAGCCAGGTAATGCGTA-3'		
CKS2^a^	NM_001827	F: 5'- CGACGACGAGGAGTTCGAGTA-3'	55	122
		R: 5'- CCTGACTCTGCTGAACACCAAG-3'		
RPL24^a^	NM_174455	F: 5'- CCGTGCAGTCAAATTCCAAA-3'	55	242
		R: 5'- CAACTCGAGGAGCAGAAACCTT-3		
FL396^a^	AY308068	F: 5'- CGCCAACACGTCTTATACCAAC-3'	55	201
		R: 5'- CTGCGAAGAGGCTTCCAATTAG-3'		
CCNB1^a^	L26548	F: 5'- CGATATGGTGCACTTTCCTCC-3'	55	145
		R: 5'- TGACCACATTCTTTGCCAGG-3'		
GAPDH^a^	BC102589	F: 5'-ACCCAGAAGACTGTGGATGG-3'	60	247
		R: 5'-ACGCCTGCTTCACCACCTTC-3'		
MSX1^b^	NM_174798	F:5'-AGAAGCAGTACCTGTCCATCG	55	382
		R: 5'-GGCCTTCTATGTCAGGTGGTA		
MSX1^c^	NM_174798	T7-F:5'- GTAATACGACTCACTATAGGGAGAAGCAGTACCTGTCCATCG	55	382
		R: 5'-GGCCTTCTATGTCAGGTGGTA		
MSX1^d^	NM_174798	SP6-R:5'- GATTTAGGTGACACTATAGAAGGCCTTCTATGTCAGGTGGTA	55	382
		F:5'-AGAAGCAGTACCTGTCCATCG		

^a ^The primer used for real-time PCR, ^b ^The primer used for DNA template amplification, ^c ^The forward primer coupled with T7 promoter (underlined) used for sense probe synthesis, ^d ^The reverse primer coupled with SP6 promoter (underlined) used for antisense probe synthesis.

### Immunohistochemistry

Eight Simmental cyclic heifers, two of each at day of estrus (day 0), day of ovulation (day 1), growth phase (day 3) and dominance phase (day 7) were slaughtered to obtain ovaries for MSX1 protein localization using immunohistochemistery. Ovaries were embedded in Tissue-Tek (Sakura Finetek Europe, Zoeterwoude, Netherlands) and snap-frozen in liquid nitrogen and stored at -80°C until sectioning. Serial sections of 5μm were cut at -20°C using rapid sectioning cryostat (Leica microsystem Nussloch GmbH, Heidelberger, Germany). The sections were mounted on poly-L-lysine coated slides (Menzel GmbH & Co. KG, Braunschweig, Germany) then washed twice in PBS for 5 min and fixed in 4% (w/v) paraformaldehyde in PBS for 45 min at room temperature. The fixed specimens were permeabilized for 5 min with 0.2% (v/v) Triton-X100 (Sigma) in PBS and washed three times with PBS. In order to inhibit non-specific binding of the antibodies, samples were subsequently blocked in 3% (w/v) bovine serum albumin (BSA) in PBS for 1 hr at 37°C. After triple washing with 0.3% (w/v) BSA in PBS slides were incubated for 15 hrs at 4°C with anti-MSX1 primary polyclonal antibody (Sigma) with a dilution of 1:100. After three consecutive washes with 0.3% (w/v) BSA in PBS, slides were further incubated for 1 hr with 1:100 dilutions of secondary anti-rabbit IgG FITC conjugated antibody (Sigma). Negative controls were processed in the same manner by omitting the primary antibody.

Iin vitro produced bovine oocytes (immature, matured) and early cleavage stages embryos (zygotes, advanced zygotes, 2-cell, 4-cell, 8-cell) were used for immunohistological localization of MSX1 protein. Ten oocytes or embryos from each stage were processed similar to ovarian sections with some modifications. Specimens were fixed in 4% (w/v) paraformaldehyde in PBS overnight at 4°C and permeabilized by 0.5% (v/v) Triton-X100 (Sigma) in PBS. In order to inhibit non-specific binding of the antibodies, samples were subsequently blocked in 3% (w/v) BSA in PBS for 1 hr. The oocytes and embryos were then incubated for 1 hr at 39°C with 1:100 dilution of anti-MSX1 primary polyclonal antibody (Sigma-Aldrich, St Louis, MO, USA). Oocytes and embryos were finally counterstained with 0.5 μg/ml propidium iodide (Sigma-Aldrich, St Louis, MO, USA) for 15 min, followed by washing with PBS. Slides were mounted with Vectashield mounting medium, covered with coverslip and viewed under confocal laser scanning microscope (CLSM LSM-510, Carl Zeiss, Germany).

### In situ hybridization

Digoxigenin-labeled riboprobes (MSX1 cDNA, sense and antisense) were generated by SP6 and T7 polymerases from linearized pGEM^®^-T Vector plasmid containing a 382 bp MSX1 cDNA insert (details of primers used Tab. 4) using Dig-labeling kit (Roche Diagnostics, Mannheim, Germany), according to the manufacturer's protocol. The same tissue samples that have been used for immunohistochemistery were serially sectioned of 5μm at -20°C as mentioned above. All sections were fixed in 4% paraformaldehyde at room temperature for 15 min. The RNA probes (50 ng/μl, sense or antisense) were added to hybridisation buffer [50% Dextran sulphate, 2.5 M NaCL, Formamide, 20× SSC, Yeast tRNA (10 mg/ml), 50× Denhardt's solution, Fish sperm DNA (10 mg/ml)], denatured at 80°C for 5 min and incubated overnight at 52°C in humidified chamber. Posthybridization washes were in 2× SSC (pH = 8), 50% formamide twice at 45°C and three times at room temperature for 10 min each. Then sections were treated with 5 μg of RNase A and 50 units of RNase T1 (Fermentas, Steinheim, Germany) in 2× SSC at room temperature for 30 min, followed by three times washing with 2× SSC at room temperature for 10 min each. After washing with TN buffer (0.1 M Tris, 0.15 M NaCl, pH 7.5) at room temperature for 5 min, the slides were incubated with 5% (w/v) blocking buffer (PerKin Elmer, Rodgau Juegesheim, Germany). Then the slides were incubated in a humidified chamber overnight at room temperature with sheep anti-DIG antibody conjugated with horseradish peroxidase (Roth, Karlsruhe, Germany), diluted to 1:100 in buffer 1 containing 1% blocking reagent. After three times washing with TNT buffer (0.05% Tween 20 in TN buffer) for 5 min each, the immunoreactions were visualized by incubating the sections with 0.02% (w/v) TSA™-Plus Fluorescent System (Perkin Elmer, Rodgau Juegesheim, Germany). Slides were counterstained with propidiumiodide (0.5 μg/ml of TNT buffer) and finally mounted with drop of Vecta shield and covered with a cover slip before viewed under fluorescence microscope.

### Brilliant cresyl blue staining

Oocytes aspirated form slaughter house ovaries were used for BCB staining. The procedure of BCB staining is done as described in our previous studies [52,53]. Briefly a total of 500 morphologically good quality immature oocytes were subjected to 26 μM BCB (B-5388, Sigma-Alderich, Taufenkirchen, Germany) diluted in mDPBS for 90 min at 38.5°C in a humidified air atmosphere. After washing the stained COCs were examined under stereomicroscope and categorized into two groups according to their cytoplasm colouration: oocytes with any degree of blue colouration in the cytoplasm (BCB^+^) and oocytes without visual blue colouration (BCB^-^). From each group, four pools of oocytes each with 50 oocytes (a total of 200) were used for mRNA isolation and subsequent real-time PCR after removal of cumulus cells as described before.

### Statistical analysis

The mRNA expression analysis for the studied genes was performed based on the relative standard curve method. The relative expression data were analysed using General Linear Model (GLM) of the Statistical Analysis System (SAS) software package version 8.0 (SAS Institute Inc., NC, USA). Differences among the mean values were tested using ANOVA followed by a multiple pair wise comparison using *t*-test. Differences of *P *≤ 0.05 were considered to be significant.

## Authors' contributions

All authors have been contributed equally to this work.
